# Letter from the Editor in Chief

**DOI:** 10.19102/icrm.2023.14116

**Published:** 2023-11-15

**Authors:** Moussa Mansour


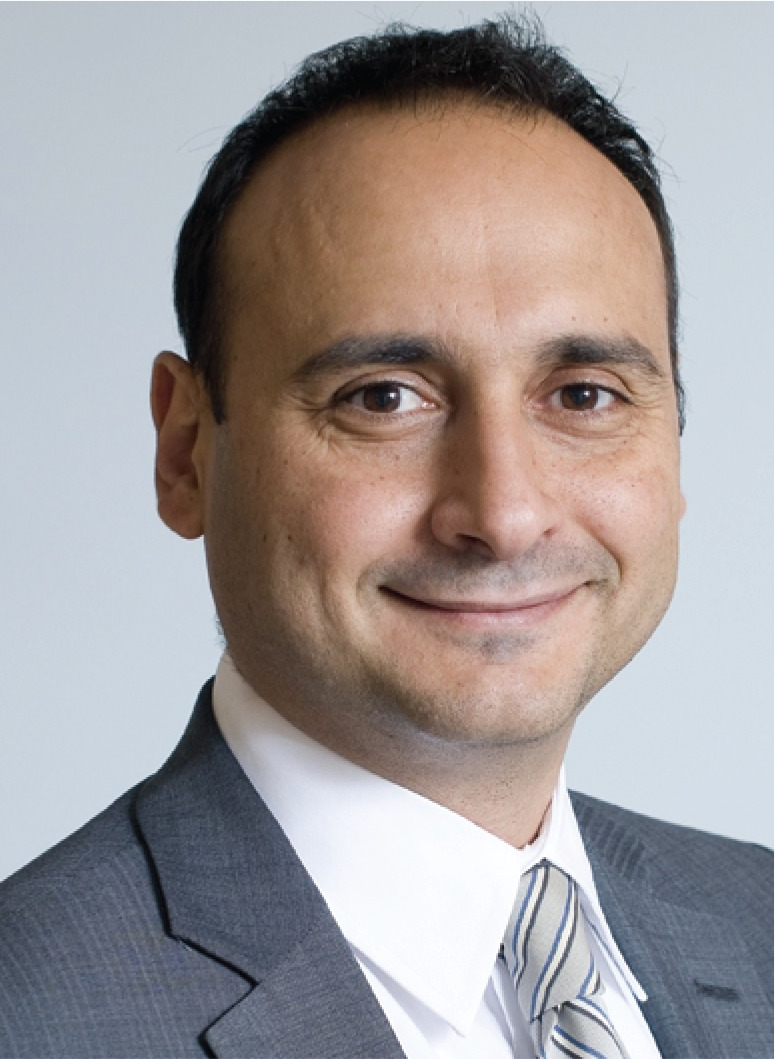


Dear readers,

One of the most common and challenging clinical situations in patients with cardiac implantable electronic devices (CIEDs) is the decision to initiate oral anticoagulation (OAC) in patients with short episodes of subclinical atrial fibrillation (AF) detected by these devices. While it has been well accepted that short episodes of AF are associated with a small increase in the risk of stroke,^1^ the initiation of OAC has been more controversial because of the bleeding risk associated with prophylactic treatment.

One study that aimed to clarify this controversy was the Apixaban for the Reduction of Thrombo-embolism in Patients with Device-detected Subclinical Atrial Fibrillation (ARTESIA) trial, which was presented at the Late-breaking Clinical Trials session at the American Heart Association meeting earlier this month, with a simultaneous publication.^2^ The study enrolled 4012 patients with CIEDs and subclinical AF lasting anywhere from 6 min to 24 h. Patients were randomly assigned to receive apixaban or aspirin. The primary efficacy and safety outcomes were the occurrence of stroke/systemic embolism and major bleeding, respectively. During a mean follow-up of 3.5 ± 1.8 years, stroke or systemic embolism occurred in 0.78% per patient-year in the apixaban group and in 1.24% per patient-year in the aspirin group (hazard ratio, 0.63; 95% confidence interval [CI], 0.45–0.88; *P* = 0.007). Separately, the rates of major bleeding were 1.71% per patient-year in the apixaban group and 0.94% per patient-year in the aspirin group (hazard ratio, 1.80; 95% CI, 1.26–2.57; *s* = 0.001). As a result, ARTESIA clearly demonstrated that the use of apixaban resulted in a lower risk of stroke or systemic embolism but a greater risk of major bleeding when compared to aspirin in patients with short episodes of subclinical AF.

The results of ARTESIA are very important but appear contradictory to the findings of the Non–Vitamin K Antagonist Oral Anticoagulants in Patients with Atrial High-rate Episodes (NOAH-AFNET 6) study^3^ published earlier this year. In NOAH-AFNET 6, 2536 patients with CIEDs and subclinical atrial high-rate episodes (AHREs) lasting longer than 6 min were randomized to receive edoxaban or placebo. The primary efficacy outcome was a composite of cardiovascular death, stroke, or systemic embolism, and the safety outcome was a composite of death from any cause or major bleeding. The trial was terminated early, at a median follow-up of 21 months, because of safety concerns and the futility of proving the efficacy of edoxaban. The incidence of stroke was approximately 1% per patient-year in both groups. A safety outcome event occurred in 5.9% per patient-year in the edoxaban group and 4.5% per patient-year in the placebo group (hazard ratio, 1.31; 95% CI, 1.02–1.67; *P* = 0.03).

The difference in results between these two studies has been elegantly explained in an editorial by Dr. Emma Svennberg.^4^ One probable reason for the difference is the low rate of stroke in the control arms in both studies compared to historical data of similar patients who have clinical AF. Another explanation is the different endpoints, with NOAH-AFNET 6 including death in both efficacy and safety endpoints, which was not the case in ARTESIA. Third, NOAH-AFNET 6 was stopped early and might have been underpowered. A longer follow-up in a larger group of patients could have led to a different result.

Based on all the published data,^1–5^ I believe it is safe to say that the increased risk of stroke associated with short periods of subclinical AF and AHREs can be reduced with OAC therapy, but this treatment is associated with an increased risk of bleeding. A personalized approach is especially important in these patients, and the unique risk of bleeding for each patient should be carefully assessed. While there is no data on the use left atrial appendage closure in patients with short episodes of subclinical AF, I believe that it should be considered as an option in high-risk patients.

I would like to end by briefly discussing the duration of subclinical AF and AHREs used in NOAH-AFNET 6, ARTESIA, and other studies. This choice was based on an earlier study showing that a 5-min cutoff excludes most episodes of oversensing.^6^ Both NOAH-AFNET 6 and ARTESIA excluded patients with episodes shorter than 6 min, which may also be clinically significant.

I hope that you enjoy reading the rest of this issue of *The Journal of Innovations in Cardiac Rhythm Management*.

Best wishes for a happy holiday season.


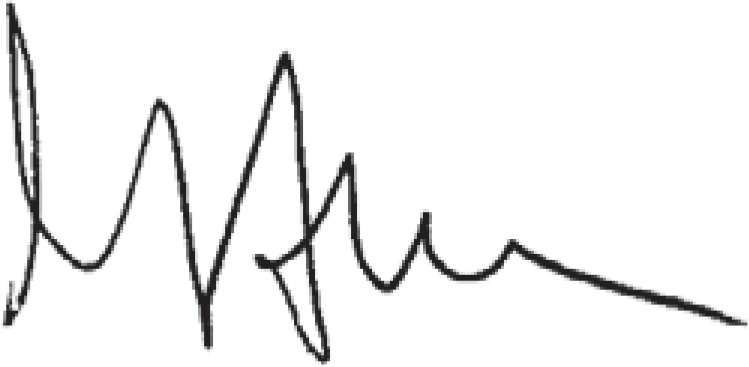


Sincerely,

Moussa Mansour, md, fhrs, facc

Editor in Chief

*The Journal of Innovations in Cardiac Rhythm Management*

MMansour@InnovationsInCRM.com

Director, Atrial Fibrillation Program

Jeremy Ruskin and Dan Starks Endowed Chair in Cardiology

Massachusetts General Hospital

Boston, MA 02114
